# Biofilm-Forming Potential of Staphylococcus aureus Isolated from Bovine Mastitis in New Zealand

**DOI:** 10.3390/vetsci5010008

**Published:** 2018-01-19

**Authors:** Shirli Notcovich, Gina DeNicolo, Steve H. Flint, Norman B. Williamson, Kristene Gedye, Alex Grinberg, Nicolas Lopez-Villalobos

**Affiliations:** 1 School of Veterinary Science, Massey University, Palmerston North 4410, New Zealand; N.Williamson@massey.ac.nz (N.B.W.); K.Gedye@massey.ac.nz (K.G.); A.Grinberg@massey.ac.nz (A.G.); N.Lopez-Villalobos@massey.ac.nz (N.L.-V.); 2 Estendart Limited, Palmerston North 4440, New Zealand; gina.denicolo@estendart.co.nz; 3 Massey Institute of Food Science and Technology, Massey University, Palmerston North 4410, New Zealand; S.H.Flint@massey.ac.nz

**Keywords:** biofilms, bovine mastitis, *Staphylococcus aureus*, *IcaA*, *IcaD*, *bap*

## Abstract

Biofilm formation is of growing concern in human and animal health. However, it is still unclear how biofilms are related to mastitis infections in dairy cattle. In this study, a comparison between two tests for biofilm formation and the association between biofilm and the presence of genes associated with biofilm formation were investigated for 92 *Staphylococcus aureus* isolates from intramammary infections. Congo red agar (CRA) and microtitre test assay (MTA) in vitro phenotypic tests were used to evaluate biofilm formation. The presence of *icaA*, *icaD*, and *bap* genes associated with biofilm formation was confirmed using the polymerase chain reaction. Results show that most of the *S. aureus* isolates, though not possessing one of the biofilm-forming genes, were able to produce biofilms. MTA was more frequently positive in identifying biofilm-forming isolates than CRA.

## 1. Introduction

Mastitis and its effects on milk quality have caused large economic losses due to the costs of animal treatment and reduced quality milk for the dairy industry over the last 50 years. In New Zealand, around 25% of clinical mastitis cases in dairy cows are due to *Staphylococcus aureus* (*S. aureus*), resulting in recurrent and/or chronic infections [1]. In many countries, *Staphylococcus aureus* is the main cause of clinical mastitis and high somatic cell counts that reduce the quality of milk submitted to processing plants [1]. 

The pathogenicity of *S. aureus* in the bovine udder is not completely understood. Lack of success in treating some chronic or recurrent infections caused by *S. aureus* could be due to the presence of virulence mechanisms that allow the microorganisms to be protected against antimicrobials and the host immune response. Biofilm formation is a virulence mechanism that protects bacteria [2]. Mastitis cases are reported to be caused by biofilm-forming strains of *S. aureus* [3,4]. Biofilms are formed in a multi-step process that involves cell attachment and formation of an extracellular matrix, which is one of the factors that protects bacteria against a hostile environment. Bacteria living in biofilms are better able to survive the host immune response and antimicrobial therapy by slowing their growth, reducing metabolism and reducing penetration of antimicrobial into the biofilm structure [5]. However, many aspects of how biofilms enable the organism to infect the udder are still poorly understood. Further research is required to address the formation of biofilms and the ability of biofilm-producing bacteria to resist antimicrobial attack. Elucidation of the mechanisms of biofilm formation by *S. aureus* may lead to new preventive or treatment measures for clinical, or subclinical, in dairy cows. 

No reports of the presence of biofilm-forming strains of *S. aureus* intramammary infections in dairy cattle in New Zealand have been found. This study aimed to examine the potential of *S. aureus* isolates from mastitis cases to form biofilm, compare methods for biofilm detection, and determine the presence of genes associated with biofilm formation.

## 2. Materials and Methods 

Isolates of *S. aureus* (*n* = 107) from a library of isolates stored at the School of Veterinary Science, Microbiology Laboratory, Massey University, Palmerston North, New Zealand, were used in this study. These isolates were obtained from bovine milk samples submitted to veterinary diagnostic laboratories. The isolates were confirmed phenotypically as *S. aureus* using biochemical tests. All the strains analysed in this study were Gram-, catalase-, and coagulase-positive. A reference strain SF01 was used as a biofilm-forming positive control. This strain was obtained from the School of Food and Nutrition, Massey University, Palmerston North, New Zealand and was originally isolated from biofilms growing on the internal surface of a stainless steel milk tank used to transport raw milk.

Congo red agar (CRA) plates were prepared following a published protocol [6]. A positive result was indicated by black colonies with a dry crystalline appearance. Non-biofilm producing colonies remained smooth, with a pearl aspect, and ranged from pink to bright red in colour. An indeterminate result was indicated by a darkening of the colonies in the absence of a dry crystalline colonial morphology. A microtitre test assay (MTA) was used to test biofilm formation in vitro following a modified version of a described method [7]. Briefly, *S. aureus* isolates were cultured overnight at 37 °C in Trypticase Soy Broth (TSB). Sterile 96-well “Flat-Bottom” plates were inoculated with 200 µL of TSB containing 0.25% glucose and 20 µL of the bacterial suspension to create a solution 1:10 and cultured statically at 37 °C for 24 h. The supernatant was then discarded and the wells were washed three times with 240 µL of sterile distilled water and air-dried at room temperature. Subsequently, the culture was fixed with 240 µL of methanol for 15 min. The liquid phase was poured off and the wells air-dried. Then, 200 µL of 1% crystal violet was added to the wells for 5 min to stain the biofilm growing on the plastic surface. After rinsing three times with distilled water, the plate was inverted and air-dried. Finally, 240 µL of glacial acetic acid was added in order to re-solubilise the stain from the biofilm. Absorbance was measured at 570 nm in a plate reader (Spectrostar Nano, BMG Labtech, Gmbh, Jena, Germany) and was used to indicate the amount of biofilm that had formed on the microtitre plate surface. Un-inoculated wells containing TSB served as blanks. Blank-corrected absorbance values for the *S. aureus* isolates were used for reporting biofilm production. 

Based on the absorbance of the bacterial films, the isolates were classified into three categories: Biofilm Negative (BN) (OD < 0.06), Slightly Positive (SP) (0.061 < OD > 0.13), and Positive (P) (OD > 0.131). The cut-off optical density (ODco) was calculated as three standard deviations (SD) above the mean of the blank [7]. Strains with OD > 2× ODco were considered P. The assay was performed in triplicate.

DNA extraction from all *S. aureus* strains was carried out using the Genaid kit for Gram-positive bacteria (Genaid, New Taipei City, Taiwan). The presence of *icaA* (188 bp), *icaD* (198 bp), and *bap* (971 bp) genes was detected by polymerase chain reaction (PCR) using forward and reverse primers for *icaA* and *icaD* as described by Mariana et al., 2009 [8] and primers for the *bap* gene as described by Cucarella et al., 2004 [9]. Primers utilised for the genes investigated were

*icaA*: Forward 5′-ACACTTGCTGGCGCAGTCAA-3′;
Reverse 5′-TCTGGAACCAACATCCAACA-3′.
*icaD*: Forward 5′-ATGGTCAAGCCCAGACAGAG-3′;
Reverse5′-AGTATTTTCAATGTTTAAAGCAA-3′.
*bap*: Forward sasp-6m 5′-CCCTATATCGAAGGTGTAGAATTGCAC-3′;
Reverse sasp-7c 5′-GCTGTTGAAGTTAATACTGTACCTGC-3′ [9].

PCR was performed in a final volume of 25 μL containing 1× PCR buffer, 1.5 mM MgCl_2_, 1 mM of each primer, 200 mM each dNTP, 1U Taq DNA polymerase (Invitrogen, Waltham, MA, USA), and 50 ng of template DNA. PCR was performed in a SensoQuest Labcycler (Göttingen, Lower Saxony, Germany) under the following conditions: an initial denaturation (95 °C for 5 min) followed by 30 cycles of denaturation (95 °C for 45 s), annealing (49 °C for 45 s), and extension (72 °C for 1 min) with a final extension of 72 °C for 7 min. PCR products (20 μL) were analysed on 1.5% (*w/v*) agarose gel stained with ethidium bromide (0.5 μg/μL) and visualised under ultraviolet transillumination and then photographed. PCR products were sent for Sanger sequencing to the Massey Genome Service (Massey University, Palmerston North, New Zealand). A PCR reaction was considered positive when positive bands of the expected sizes were observed on gels. DNA sequences from all samples were trimmed and aligned to the reference sequences accession numbers AF086783 and AF288402 downloaded from Genbank using Geneious v 6.0 (http://www.geneious.com).

## 3. Results and Discussion

From the 107 isolates analysed, 15 negative to the coagulase test were removed from the study. Ninety-two *S. aureus* isolates were tested phenotypically for biofilm formation. 

The MTA showed that most of the 92 isolates had the ability to produce biofilm in tissue culture plates. Positive and SP results comprised 93.4% (86/92) of the isolates (Table 1). A chi-square test showed significantly more positive results when using MTA than CRA tests (*p* < 0.001). 

The PCR results showed that all but one of the isolates were positive for both *icaA* and *icaD* genes, with the one exception being negative for *icaA*. Sequencing of PCR products from two isolates confirmed the identity of the amplicons as *icaA* and *icaD* genes. Compared to the reference sequence (AF086783), a 12 bp deletion was found in the *icaD* sequence from the two sequenced amplicons. The sequence from the isolate that produced the 12 bp deletion was identical to numerous previously published *S. aureus* sequences, including LT671859, which was originally isolated from an infected human wound [10]. The *bap* gene was not identified in any of the isolates tested. Many genes are involved in biofilm synthesis. Genes such as biofilm-associated protein (*bap*), bone-sialoprotein-binding protein (*bbp*), clumping factor A and B, collagen-binding protein, elastin-binding protein S (*ebpS*), and intercellular cell attachment A and D (*icaA* and -*D*) have been shown to play a role in biofilm synthesis. However, in *S. aureus* strains, biofilm synthesis is mainly encoded by *ica, bap*, and *agr* although other genes may be involved [11,12].

The results obtained in this study showed no association between the synthesis of biofilms in the phenotypic studies and the presence of *icaA*, *icaD*, and *bap* genes coding for biofilm synthesis. Most of the isolates were *icaA-* and *icaD*-positive and *bap*-negative. Despite the lack of the *bap*-coding gene for biofilm formation in the *S. aureus* strains tested, 93% of them formed biofilms. These results suggest that, in accordance with other studies, *ica* and *bap* represent alternative synthetic pathways for biofilm production [13]. In previous studies, the *bap* gene has been found in pathogenicity islands that are able to be transmitted horizontally between strains and are not present in every strain [13,14]. Loss of these islands by horizontal gene transfer may explain why the isolates in this study were negative for the *bap* gene. Another reason for the absence of the *bap* gene in this study may be that the strains analysed originally belonged to human *S. aureus* infections. *Staphylococcus aureus* transmission between humans and bovine does occur although at low rates [15,16]. The 12 bp deletion found in the *icaD* gene sequenced in this study is identical to a deletion found in *S. aureus* strains isolated from human wounds, e.g., LT671859. In other studies, the *bap* gene was found in 5% of bovine isolates of *S. aureus* and was absent in all the human isolates analysed [11]. Although the isolates in this study were all from bovine clinical mastitis, the phylogeny of these strains is unknown. Due to time and resource limitations, testing of this hypothesis was not possible in this research. In contrast to the current results, in other studies, the *bap* gene was found in 95.6% of the bovine mastitis isolates [4]. The ability to form a biofilm is highly influenced by the environment in which bacterial colonies develop [6]. Strain SFO1, which was a strong biofilm producer in studies carried out on stainless steel in a milk environment, was, in this study, an undetermined biofilm producer in the CRA test and SP for the MTA. However, PCR showed that this strain (SFO1) contained two of the three genes related to biofilm production (*icaA* and *icaD*), suggesting that the different environments had influenced the expression of the *ica* genes for biofilm synthesis. It has been reported that a *Staphylococcus aureus* bacterial strains (e.g., V329), experimentally inoculated into a mammary gland, varied from biofilm-forming to biofilm-negative in a process known as phase variation [17,18]. It is believed that this could be a mechanism to adapt to the environment and may explain the phenomenon observed in this study with strain SFO1 varying from strongly biofilm positive to slightly positive. The expression levels of these genes may have varied between these studies. The presence of the *ica* locus in bacteria isolated from chronic staphylococcal infections in other species suggests that the *ica* locus is highly conserved within *Staphylococcus* spp. [19,20]. Biofilm formation is a resistance mechanism present in bacterial strains causing bovine mastitis around the world [3,4]. The results of this study show that New Zealand is no exception. However, elucidation of the expression of genes and phase variations during the infection process is still required. 

## 4. Conclusions

Most *S. aureus* isolates in this study were able to produce biofilms despite being negative for one of the biofilm-forming genes. The MTA identified more biofilm-forming strains than CRA. Further studies on biofilm formation in the mammary gland are necessary, as well as the development of means to prevent or treat any biofilm-associated infections.

## Figures and Tables

**Table 1:** Summary of the results of tested isolates.

Test Results	Positive MTA	Slightly Positive MTA	Negative MTA	Total
Positive CRA	16	9	0	25
Negative CRA	2	14	4	20
Indeterminate CRA	18	27	2	47
Total	36 *	50 *	6	92

* means (*p* < 0.05) difference in sensitivity of the MTA analysis when compared with CRA.

## References

[1] Petrovski K.R., Heuer C., Parkinson T.J., Williamson N.B. (2009). The incidence and aetiology of clinical bovine mastitis on 14 farms in northland, New Zealand. N. Z. Vet. J..

[2] Melchior M.B., Fink-Gremmels J., Gaastra W. (2006). Comparative assessment of the antimicrobial susceptibility of *Staphylococcus aureus* isolates from bovine mastitis in biofilm versus planktonic culture. J. Vet. Med. B Infect. Dis. Vet. Public Health.

[3] Szweda P., Schielmann M., Milewski S., Frankowska A., Jakubczak A. (2012). Biofilm production and presence of *ica* and *bap* genes in *Staphylococcus aureus* strains isolated from cows with mastitis in the eastern poland. Pol. J. Microbiol..

[4] Hensen S.M., Pavičić M., Lohuis J., De Hoog J., Poutrel B. (2000). Location of *Staphylococcus aureus* within the experimentally infected bovine udder and the expression of capsular polysaccharide type 5 in situ. J. Dairy Sci..

[5] Stewart P.S., William Costerton J. (2001). Antibiotic resistance of bacteria in biofilms. Lancet.

[6] Freeman D.J., Falkiner F.R., Keane C.T. (1989). New method for detecting slime production by coagulase negative staphylococci. J. Clin. Pathol..

[7] Oh S.-W., Chen P.-C., Kang D.-H. (2007). Biofilm formation by enterobacter sakazakii grown in artificial broth and infant milk formula on plastic surface. J. Rapid Methods Autom. Microbiol..

[8] Mariana N., Salman S., Neela V., Zamberi S. (2009). Evaluation of modified congo red agar for detection of biofilm produced by clinical isolates of methicillin resistance *Staphylococcus aureus*. Afr. J. Microbiol. Res..

[9] Cucarella C., Tormo M.A., Ubeda C., Trotonda M.P., Monzon M., Peris C., Amorena B., Lasa I., Penades J.R. (2004). Role of biofilm-associated protein bap in the pathogenesis of bovine *Staphylococcus aureus*. Infect. Immun..

[10] Mannala G.K., Hain T., Spröer C., Bunk B., Overmann J., Alt V., Domann E. (2017). Complete genome and plasmid sequences of *Staphylococcus aureus* edcc 5055 (dsm 28763), used to study implant-associated infections. Genome Announc..

[11] Cucarella C., Solano C., Valle J., Amorena B., Lasa Í., Penadés J.R. (2001). Bap, a *Staphylococcus aureus* surface protein involved in biofilm formation. J. Bacteriol..

[12] Melchior M.B., van Osch M.H.J., Graat R.M., van Duijkeren E., Mevius D.J., Nielen M., Gaastra W., Fink-Gremmels J. (2009). Biofilm formation and genotyping of *Staphylococcus aureus* bovine mastitis isolates: Evidence for lack of penicillin-resistance in agr-type ii strains. Vet. Microbiol..

[13] Tormo M.A., Knecht E., Gotz F., Lasa I., Penades J.R. (2005). Bap-dependent biofilm formation by pathogenic species of staphylococcus: Evidence of horizontal gene transfer?. Microbiology.

[14] Úbeda C., Tormo M., Cucarella C., Trotonda P., Foster T.J., Lasa Í., Penadés J.R. (2003). Sip, an integrase protein with excision, circularization and integration activities, defines a new family of mobile *Staphylococcus aureus* pathogenicity islands. Mol. Microbiol..

[15] Grinberg A., Hittman A., Leyland M., Rogers L., Le Quesne B. (2004). Epidemiological and molecular evidence of a monophyletic infection with *Staphylococcus aureus* causing a purulent dermatitis in a dairy farmer and multiple cases of mastitis in his cows. Epidemiol. Infect..

[16] Smyth D.S., Feil E.J., Meaney W.J., Hartigan P.J., Tollersrud T., Fitzgerald J.R., Enright M.C., Smyth C.J. (2009). Molecular genetic typing reveals further insights into the diversity of animal-associated *Staphylococcus aureus*. J. Med. Microbiol..

[17] Tormo M.A., Ubeda C., Marti M., Maiques E., Cucarella C., Valle J., Foster T.J., Lasa I., Penades J.R. (2007). Phase-variable expression of the biofilm-associated protein (bap) in *Staphylococcus aureus*. Microbiology.

[18] Baselga R., Albizu I., De La Cruz M., Del Cacho E., Barberan M., Amorena B. (1993). Phase variation of slime production in *Staphylococcus aureus*: Implications in colonization and virulence. Infect. Immun..

[19] Nemati M., Hermans K., Devriese L.A., Maes D., Haesebrouck F. (2009). Screening of genes encoding adhesion factors and biofilm formation in *Staphylococcus aureus* isolates from poultry. Avian Pathol..

[20] Singh A., Walker M., Rousseau J., Weese J.S. (2013). Characterization of the biofilm forming ability of staphylococcus pseudintermedius from dogs. BMC Vet. Res..

